# Association between Multiple Recurrent Events with Multivariate Modeling: A Retrospective Cohort Study

**Published:** 2018-12-23

**Authors:** Freshteh Osmani, Ebrahim Hajizadeh, Ali Akbar Rasekhi

**Affiliations:** ^1^ Department of Biostatistics, Faculty of Medical Sciences, Tarbiat Modares University, Tehran, Iran

**Keywords:** Breast Cancer, Multivariate Analysis, Recurrent Events

## Abstract

**Background:** Recurrent event data are often encountered in biomedical research, for example, recurrent infections or recurrent hospitalizations for patients after renal transplant. In many studies, there are more than one type of events of interest. We aimed to identify the association between two types of events using multivariate joint modeling and then apply this statistical method in the clinical data set.

**Study design:** A retrospective cohort study

**Methods:** Overall, 342 subjects with breast cancer whose records were registered for follow-up in a Cancer Research Center at Shohadaye Tajrish Hospital, Tehran, Iran from 2006 to 2015 were investigated. These patients were monitored for at least 6 months after diagnosis and their latest status were recorded. Joint frailty model was used for modeling the relationship between two types of recurrences with Frailty package in R software.

**Results:** When the terminal event was considered as death, three-year and five-year survival rates for the patients were 0.79 and 0.68, respectively. Given the results obtained from a fitted joint frailty model, the risk of multiple recurrences (local and metastases) increased for the patients with tumor grades greater than I.

**Conclusion:** With regard to the significant variance of the frailty component of the metastases event, it can be inferred that patients with the same predictive variables are prone to different levels of metastases risk and, on the other hand, given the low frequency of types of recurrences, caution should be exercised when considering the obtained results.

## Introduction

In the survival analysis literature, different methods were proposed for estimating recurrent events of a type. Conditional models^1^ and marginal models^2^ were all presented for analyzing recurrent events of a type. These models are based on modeling a hazard function. Despite the advancement in methods of analyzing data on recurrent events of a type, methods used to analyze recurrent events of several types, including non-homogeneous Poisson’s processes using random and fixed effects with inferential procedures, are all based on the maximum likelihood estimation. Such parametric methods require identifying the correctness of the considered in-person correlation structure which is difficult to achieve for multiple recurrent events. Semi-parametric robust methods are desirable when a correlation structure is not considered. Marginal regression models for multivariate failure time data proposed by Spiekerman and Lin^3^ (and Aalen, Borgan, and Gjessing^4^ can be generalized to the analysis of multiple recurrent events^5^.

Breast cancer after skin cancer is the most prevalent cancer among women^6^. Besides the high prevalence rate of breast cancer among Iranian women, the facts that 16% of all cancers in Iran are related to breast cancer and that Iranian women, compared to developed countries, developing this disease at least a decade earlier^7^ double the importance of studying this issue. One of the possible complications of breast cancer after conducting a treatment (surgery) is either metastases or local recurrence of the disease. The primary causes of death in breast cancer are tumor invasion and metastases^8,9^. Despite significant advances in the diagnosis and treatment of breast cancer, a high mortality rate and incidence of metastases breast cancer in affected women treated by performing a surgery and applying necessary treatments still remain as medical issues^10^.

Patients with metastases had shorter lifespans compared to other patients^6^. On the other hand, metastatic recurrence of the disease decreases a patient’s physical and mental dimensions of quality of life. Therefore, recognizing factors affecting the incidence of metastases and examining the relationship between the two types of recurrences, i.e. metastases and local recurrences, among patients with breast cancer is very important in the process of identifying and treating this disease^11^. Different factors affecting the incidence of metastases have been studiesalready^12^. However, objectives related to analyzing such recurrent events can include describing the status of recurrent events in people, changing the recurrence rate of a process from a person to another person, and investigating the relationship of fixed dependent variables or time-dependent variables with the occurrence time of recurring events. These objectives can be verified by models associated with analyzing recurrent events. Due to their ability to conduct a simultaneous investigation of two processes of recurrent events and to achieve unbiased and efficient estimates for parameters, in survival analyses, joint models are applied for analyzing follow-up studies including carrying out a simultaneous examination of two survival events^13^.

The simplest way of analyzing recurrent events is to only consider the first occurrence for each patient and use Cox’s proportional hazards model for just one of the events. This is a direct method and avoids considering complexities such as the effect of the first event on the risk of the occurrence of the next event. Furthermore, only considering the first event is not satisfactory for evaluating the natural history of the disease and examining the effects of therapeutic interventions. Additionally, by the sole consideration of the first event, this kind of analysis does not apply all available data and does not correctly estimate all advantages of performing a therapeutic approach.

Various models have been presented to fit data on recurrent events, which are all essentially generalizations of the Cox’s proportional hazards model. In addition, in studies where several failures occur during the follow-up period, there are always individual factors, which create a correlation between the time of the occurrence of events in the same people and the reason for the differences amongst people (dispersion amongst individuals). Standard survival models, like the Cox’s proportional hazards model, ignore the effects of unobserved individual factors and lead to an incorrect estimation of model parameters. In recent studies, a random component was used to express these unknown factors and correlations amongst the incidence of recorded events in a patient. This random component is known as frailty^16^. Accordingly, joint modeling is suitable for such a condition since it is able to study two processes at the same time and achieve unbiased and efficient estimates for parameters to analyze several follow-up studies^13^. On the other hand, people may be exposed to more than one recurrent event during their lifetime with the disease. Since data related to recurrent events have existed in most medical longitudinal studies and, sometimes, more than one type of recurrent events have been considered in these studies, a multivariate joint frailty model was proposed in the current study for analyzing such data and examining hazard functions of various types of recurrent events with respect to the effect of frailty. The complexity of the mentioned modeling was that it simultaneously modeled two types of recurrences, i.e. local and metastases recurrences.

## Methods

This study was a retrospective cohort study, in which all patients with breast cancer referred to Shohadaye Tajrish Hospital, Tehran, Iran from 2006 to 2015 were considered as a statistical population. The current study was extracted from an Phd thesis, which was checked and approved by the Ethics Committee of the Tarbiat Modares University of Medical Sciences (IR.TMU.REC.1396.632).

During the abovementioned years, patients with definite breast cancer diagnoses were enrolled and examined in a cancer ward at this hospital as a historic cohort. These patients’ data were collected by the Cancer Research Center of this hospital under the supervision of Shahid Beheshti University of Medical Sciences. Data needed to carry out the present study were extracted from the patients’ medical records and their latest conditions in terms of the recurrence of the disease recorded by this center. An inclusion criterion was considering all patients with definite breast cancer diagnoses who had been followed up at Shohadaye Tajrish Hospital for at least 6 months after their surgeries and an exclusion criterion was regarding cases whose records were incomplete and patients who had been followed up to five months that it means that patients who had less than 6 months follow up were excluded from the study. Finally, by eliminating variables overlapped with the results of the current study, the final sample size was considered 342 patients.

In this study, variables under study were age, diagnosis time, family history of breast cancer, tumor size, levels of the involvement of lymph node removed after surgery, metastases, surgical type, tumor grade, estrogen receptor levels, progesterone receptor levels, undergoing a chemotherapy, the stage of the disease are considered as independent variables and the time until local and metastases recurrences are considered as dependent variables. In this historic cohort study, all patients with definite pathologic diagnoses whose records could be used were enrolled. Alive patients who did not experience any local and metastases at the end of the study and patients who, after a certain period of time, provided no data about their survival status were considered as right-censoring.

In the current study, Using Kaplan-Meier estimator, a median of disease-free survival time (the time till the occurrence of the first surgery is known as disease-free survival time) for breast cancer patients was estimated. The following additive joint frailty model was used for modeling joint recurrent events.

=h_1i_(t_1ij_)exp(β^T^_1_Z^(L)^_i_+ɵ_1i_)

=h_2i_(t_2ij'_)exp(β^T^_2_Z^(D)^_i_+ɵ_2i_)

In this model, h_1_(t), h_2_(t) are basic hazard functions for local and metastases recurrences in i^th^person, respectively. Moreover, Z^(L)^ and Z^(D)^ are auxiliary vectors for local and metastases recurrences, respectively. Furthermore, β^T^_a_ , β^T^_a_ are vectors for corresponding regression parameters. Parameters specific to each part are bivariate frailties logarithms {(ɵ_1i_,ɵ_2i_)}^n^_i=1_ which are patient-specific. In this regard, ɵ_1i_,ɵ_2i_ indicate frailties. These frailties can demonstrate that patients with higher levels of frailty, compared to others, are more prone to the risk of recurrence. The effects of ɵ_1i_ and ɵ_2i_ work on the time of recurrence type 1 (T_1_ ) and the time of recurrence type 2 (T_2_ ). Hence, it was assumed that effects of these two types of recurrences were not the same. Finally, a bivariate normal distribution for {(ɵ_1i_,ɵ_2i_)} was presented as follows:

$$N\left(\left(\begin{array}{l} 0\\ 0 \end{array}\right)\right),\left(\begin{array}{l} \sigma_{1}^{2}\;\rho \sigma_{1}\sigma_{1}\\ \rho \sigma_{1}\sigma_{2}\;\sigma_{2}^{2} \end{array}\right)$$

Hence, the variance of ui (θ) specifies the dependency between occurrences of the recurrent events of type 1 and the survival and also the interrecurrence dependency. Similarly, the variance of vi (η) specifies the dependency between occurrences of the recurrent events of type 2 and the survival and also the interrecurrence dependency. In the current study, the proposed model was run by the frailtypack in R software version 3.4.1 and estimating the parameters was done by using maximum penalized likelihood estimation.

## Results

A total of 342 patients were enrolled. Univariate and multivariate analyses were conducted on these patients. In the current cohort study, a corpus of 342 women with breast cancer was examined. The patients aged 22 to 84 yr with a mean age of 47.84, a standard deviation of 11.75 yr, and a median of 47 years. The median follow-up time was 113 months. Among these 342 patients under study, 87 patients (25.4%) experienced the incidences of recurrences and the other 225 patients (74.6%) did not experience such recurrences. 44 people (12.9%) suffered from stage 1 of the disease, 168 people (49.1%) suffered from stage 2 of the disease, 121 people (35.4%) suffered from stage 3 of the disease, and only 9 people (2.6%) suffered from stage 4 (the most dangerous stage of this disease).

In these patients, the median disease-free survival time was 30.57 months with a minimum of 6 months and a maximum of 187 months. A distribution of the frequency of the independent variables under study was presented in Table 1.

**Table 1:** The distribution of the frequency of characteristics among the patients with breast cancer

**Variables**	**Number**	**Percent**
Family history		
None	233	68.1
Immediate family member	52	15.2
Extended family member	57	16.7
Tumor size (mm)		
<2	72	21.1
2-5	211	61.7
>5	59	17.3
Stage of the disease		
Stage 1	44	12.9
Stage 2	168	49.1
Stage 3	121	35.4
Stage 4	9	2.6
Chemotherapy		
Yes	330	96.5
No	12	3.5

Using Kaplan-Meier estimator, a median of disease-free survival time (the time till the occurrence of the first recurrence after surgery is known as disease-free survival time) for breast cancer patients was estimated to be 30.57 and disease-free one-year, three-year, and 5-year survival rates were 96% (0.01), 79% (0.026), and 68% (0.033), respectively.(Table 2)

**Table 2:** The disease-free one-year, three-year, and 5-year survival rates of the patients with breast cancer

**Follow-up time (yr)**	**Survival probability**	**Standard error**
1	0.96	0.010
3	0.79	0.026
5	0.68	0.033

In this section, the joint frailty model was fitted with an approximated basic hazard function using smoothing methods. The maximum penalized likelihood estimation was applied for estimating the parameters. The maximum likelihood estimation was used for models with a fixed basic hazard function. Given the results obtained from the fitted model, the risk of local and metastases recurrences was higher in the patients with at least one N+ lymph node and/or in the patients with a tumor grade of greater than 1 (HR>1). Moreover, no significant difference was found in terms of the risk of death between the patients who were younger than 40 yr old and those who were older than 60 yr old. However, there was a slightly significant difference in hazard ratio between the patients aged 40 to 60 yr old and the patients older than 60 yr old. The risk of multiple local and metastases recurrences was higher in the patients who were younger than 40 yr old compared to the patients who were older than 60 yr old. Furthermore, the tumor size (>20 mm) had a significant effect on the risk of recurrences (*P*<0.05). The risk of local recurrence was higher among the patients with HER2+. Besides, the hazard of metastases recurrence was higher for subjects with HER2+ (HR>1). (Table 3)

**Table 3:** Results of the joint frailty modeling for multiple recurrent events, local recurrence, and metastases recurrence in the breast cancer patients

**Local recurrence**	**HR**	**95% CI**
Age (yr)		
>60	1.00	
≤40 years old	2.86	1.76, 4.64
40-60 years old	1.32	0.94, 1.86
Tumor grade		
I	1.00	
II	2.79	1.53, 5.09
III	4.79	1.33, 3.17
Tumor size (mm)		
<20	1.00	
>≥20	1.61	1.15, 2.25
HER2+		
Negative	1.00	
Positive	1.83	1.18, 2.82
**Metastases**	**HR**	**95% CI**
Age (yr)		
>60	1.00	
≤40 years old	2.81	1.31, 6.03
40-60 years old	0.80	0.49, 1.29
Tumor grade		
I	1.00	
II	1.63	1.15, 2.30
III	4.56	2.26, 9.20
Tumor size (mm)		
<20	1.00	
≥20	5.92	2.53, 13.86
HER2+		
Negative	1.00	
Positive	2.19	1.10, 4.34
θ = var(ui ) (SE)	1.10	0.11
η = var(vi) (SE)	7.39	0.63

## Discussion

In the present study, in order to simultaneously consider two recurrent events and to achieve more accurate results, the joint frailty model was used for modeling multiple recurrent events and the maximum penalized likelihood estimation was applied for estimating the hazard functions. This approach is also consistent with informative censoring of recurrent events. The proposed model showed that there was a correlation between the two types of recurrences. This method can also cover the relationship between recurrent events and the terminal event^14^. This method is better for practical situations and is better and more efficient compared to applying two separated models (having a separate joint frailty model for each type of recurrences). On the other hand, this method also considers the correlation between events and unobserved heterogeneity in data. As inferred from the concept of joint frailty models, a joint frailty component can improve the fitness of a model. In addition, ignoring it when fitting a model can cause an impairment in the fitness of the model. This approach accounts for between events dependencies, interrecurrence dependencies, and nonobserved heterogeneity. As observed in Putter and van Houwelingen in the context of shared frailty models, the latent frailties can pick up anything that improves the fit of the model^15^. This could be true heterogeneity but also lack of fit of the model without frailties. For instance, the lack of fit of the PHs models can be confused with the presence of heterogeneity, that is, it is difficult to tell whether there is true heterogeneity or whether the PHs assumption is violated^16^.

We also did the PHs assumption conditionally on frailties in our proposed model. Another advantage of this proposed model is that the effects of various covariates are evaluated by two types of hazard functions. These variables can be independent of time and/or time-dependent. In the application of these models, we concluded that the risk of locoregional recurrences is associated with the risk of metastatic recurrences and the risk of metastatic recurrences is also associated with death. The risk of death is not directly associated with the risk of locoregional recurrence. However, Wapnir et al showed a dependence between locoregional relapses and death^17^. A major issue of debate in this area of research is whether for death following locoregional relapse, the locoregional relapse is causal (produces additional risk of metastatic spread) versus incidental (in fact, a marker or flag for high-risk disease that may have disseminated already at the time of diagnosis^18-20^. For example, the reason early locoregional relapse associated with death is that it is indeed a flag for aggressive^21^, already disseminated disease (and also greater risk of locoregional relapse concurrently), whereas a late locoregional relapse may be just a de novo recurrence found early due to increased screening vigilance, and any disseminated disease has just begun and will not increase mortality risk any time soon.

Generally, there are several reasons for using frailty models to provide time-to-recurrence responses, including achieving an overall possible correlation by applying a bias correction for a regression coefficient in survival analysis and whether one or both types of recurrences can be used as an alternative endpoint. In cases where there was a significant difference in a subgroup of patients, a frailty model can be used to evaluate such heterogeneity in the population under study^22^. A number of authors have also applied the frailty model to analyze data on breast cancer^23^.

In the present study, the mean age of the patients was 47.81 yr, which is consistent with findings of other studies carried out in Iran reporting that the mean age of breast cancer patients was between 45 and 50 years^24,25^. The age distribution of women with breast cancer in Iran indicates that the diagnosis age is lower in Iran compared to that in Western Europe and North America. This means that Iranian women are more likely to suffer from this disease earlier. This was confirmed by a number of studies examined the issue in Iran^24,25^. The median disease-free survival time in this study was 64 months and the five-yr survival rate for the patients was 68%. In another study^23^, a disease-free 5-yr lifespan was reported as 77.3% (21). HER+2 in patients was not considered as a prognostic factor for metastases recurrence in the frailty model; however, it can be regarded as a prognostic factor in the recurrence of metastases^26^. The degree of tumor malignancy was recognized as a significant factor in the prognosis of local and metastases recurrences in the patients, which is in line with other results^25^showing that patients with the first-degree malignancy had more survival rate than patients with the second-degree and third-degree malignancy. Moreover, during the follow-up period of the patients under study, none of the two events, i.e. death or metastases, was observed in the patients with the first-degree malignancy. The tumor size was proved to be a prognostic factor for patients’ survival rate ^27^. In the current study, this variable was also significant in the joint model for two types of local and metastases recurrences. The variance in the distribution of frailty in the above model for local recurrence was 1.1, which did not differ significantly with the value of 1 and for metastases recurrence, this value was 7.39, which indicated that only considering the effect of auxiliary variables did not determine the state of recurrence of the disease and individual characteristics were also effective in the recurrence of tumors. In other words, the effect of unknown variables or variables that are not included in the model was also important in predicting metastases recurrences of the disease and these factors, a small part of which are individual characteristics of patients, play significant roles in predicting the patients' statuses. In the Gohari's study ^20^ the variance of frailty was 0.31 and in the study of carried out by Rondeau^23^, it was 0.35. This suggested a high degree of non-homogeneity amongst breast cancer patients participating in this study compared to those participating in the studies conducted earlier^22,25^.

## Conclusion

Even the patients with the same explanatory variables presented different risks of metastases recurrence. Similarly, in a sample selected from a large population of women with breast cancer, it was inferred that there was a positive relationship between multiple recurrences among the patients. In other words, the presented model used in the current study for modeling the dataset converged correctly. In this study, a small number of both types of local and metastases recurrences were observed. Therefore, the random effects of u_i_ and v_i_ reflected the relationship between the two types of recurrences rather than the interpersonal dependence of the patients. In this particular case, achieving the independence of the two answers (multiple recurrences) may be difficult. Since this study included a small number of recurrent events, cautions should be exercised when considering the obtained data.

## Acknowledgements

The authors would like to thank the Breast Cancer Research Center of Shohadaye Tajrish Hospital for providing access to the data on the patients with breast cancer. This article was extracted from a doctoral thesis presented at the Faculty of Medicine, Tarbiat Modares University. In this way, the authors sincerely thank faculty members and employees of the Faculty of Medicine at Tarbiat Modares University.

## Conflict of interest statement

There is no conflict of interest in this study.

## Funding

This work was supported by Tarbiat Modares University, faculty of medical science. The collection of cancer data used in this study was supported by Shahid Beheshti Breast Cancer Research Center in Iran.

## Highlights

- Breast cancer patients with the same predictors are prone to different levels of metastases risk.
- By ignoring the relationship between multiple recurrences in the patients with breast cancer, a significant correlation was missed.
- According to the results, a simultaneous examination of different types of recurrences leads to the achievement of more accurate results
